# Maintaining long-term frequent tea consumption could reduce the risk of cognitive decline: results from a 10-year longitudinal study

**DOI:** 10.3389/fnut.2025.1569850

**Published:** 2025-06-18

**Authors:** Jie Huang, Hongfen Wang, Yuying Wang, Jiaqi Xu, Jiameng Ni, Mengjie Li, Bin Chen, Zaixiang Tang, Yu Zhang, Liping Tan

**Affiliations:** ^1^The Second Affiliated Hospital of Soochow University, Department of Biostatistics, School of Public Health, Suzhou Medical College of Soochow University, Suzhou, Jiangsu, China; ^2^Jiangsu Key Laboratory of Preventive and Translational Medicine for Major Chronic Non-Communicable Diseases, MOE Key Laboratory of Geriatric Diseases and Immunology, Suzhou Medical College of Soochow University, Suzhou, Jiangsu, China; ^3^Department of Neurology, The First Center of the PLA General Hospital, Beijing, China; ^4^Suzhou National New and Hi-tech Industrial Development Zone Center for Disease Control and Prevention, Suzhou, Jiangsu, China; ^5^School of Urban Governance and Public Affairs, School of Wellness Industry, Suzhou City University, Suzhou, Jiangsu, China

**Keywords:** tea, frequency, habits, cognitive function, inverse probability of treatment weighting

## Abstract

**Background:**

Previous studies have suggested that tea may have neuroprotective effect. This study aimed to investigate the association between tea consumption frequency and cognitive decline, taking into account changes in consumption habits and selection bias.

**Methods:**

This study used data from the Chinese Longitudinal Healthy Longevity Survey (CLHLS) in 2008, 2011, 2014, and 2018 years. Changes in tea consumption habits were identified based on the frequency and consistency of tea consumption at baseline and at the last follow-up. Cognitive function was assessed by using Mini-Mental State Examination (MMSE). Cox proportional hazards models were conducted to estimate association between tea consumption habits and cognitive decline. Inverse probability of treatment weighting (IPTW) was applied to reduce selection bias.

**Results:**

Consistently frequent tea consumption reduced the risk of cognitive decline, whereas inconsistently frequent tea consumption did not. Compared with consistently infrequent tea drinkers, the multivariable-adjusted HR of cognitive decline was 0.98 (95% CI: 0.90, 1.06) for inconsistently frequent tea drinkers, and 0.86 (95% CI: 0.76, 0.96) for consistently frequent tea drinkers. With increasing frequency of tea consumption, the risk of developing cognitive decline decreased (*P* for trend: 0.023). Similarly, the association remained robust after IPTW adjustment. Consistently frequent tea drinkers had a 12% (HR:0.88, 95% CI: 0.77, 0.99) reduced risk of cognitive decline, whereas no significant association was observed for inconsistently frequent tea consumption (HR:0.98, 95% CI:0.91, 1.07) (*P* for trend < 0.001). Additionally, frequent consumption of both tea and fruit has a synergistic effect on cognitive health (*P* for interaction = 0.041).

**Conclusion:**

Maintaining an uninterrupted habit of frequent tea consumption over time could reduce the risk of cognitive decline, whereas inconsistently frequent tea consumption was not significant, even after IPTW adjustment. Regular consumption of tea and fruit has a synergistic effect on cognitive health.

## 1 Introduction

The decline in cognitive function leading to dementia has become one of the leading causes of disability and death among older individuals worldwide (1, 2). A recent study suggested the number of people with dementia worldwide will increase from 57.4 million cases in 2019 to 152.8 million in 2050 (3). Due to the complexity and heterogeneity of dementia, treatment options are limited (4). Therefore, preventing and delaying the development of the disease in its early stages is particularly crucial. Cognitive decline and mild cognitive impairment, as potential precursors of dementia (such as Alzheimer’s disease), can present early clinical indications (5). In recent years, a body of accumulating evidence has indicated a correlation between dietary habits and cognitive function (6).

Tea originated in China and has become popular all over the world. Tea contains a number of bioactive components with health benefits, such as catechins (especially epigallocatechin gallate [EGCG]), theaflavins, theanine, and caffeine (7). The neuroprotective mechanisms of tea are complex, with catechins generally considered to be the key compounds. *In vivo* and *in vitro* studies have shown that EGCG crosses the blood–brain barrier (8, 9). Long-term administration of catechins prevented oxidative stress-related brain aging in mouse models (10). At the same time, a study has suggested that long-term administration may prevent age-related spatial learning and memory decline in mice by regulating hippocampal cAMP-response element binding protein signaling cascade (11). In addition, theanine is a unique amino acid in tea that can exert neuroprotective effects by inhibiting glutamate receptors in rat models (12).

However, population-based prospective studies on the association between tea consumption and cognitive decline are still underdeveloped. Fewer than 10 prospective studies have been conducted and reported contrary results (6, 13). Two of them from China (14, 15), two from the Japan (16, 17), and one each from Singapore (18), the United States (19), and Germany (20). A study from Singapore suggested that tea consumption was associated with a lower risk of cognitive decline, and a dosage-response relationship existed (18). Two Japanese studies both showed green tea was associated with lower risk of cognitive decline (16, 17). In contrast, a prospective study conducted in eastern China showed that tea consumption was not associated with cognitive decline (15). Similarly, no association was found between green or black tea and cognitive decline among older adults in Germany (20) or the United States (19).

Frequency and duration of consumption were important factors influencing tea effects. However, tea drinking habits may change over time. Most previous studies have considered only baseline tea consumption information, ignoring the potential impact of longitudinal changes on cognitive function. This may not reflect their substantive associations. Therefore, a more refined assessment of cumulative exposure is more accurate for clarifying the causal relationship between tea consumption and cognitive decline. In addition, inverse probability of treatment weighting (IPTW) is a method based on propensity scores. The propensity score methods aim to approximate randomization (i.e., pseudo-randomization) by compensating for the different probabilities of participants being assigned to exposure, which could reduce the bias in estimating treatment effects and the likelihood of confounding in non-randomized observational data (21, 22). IPTW provides an unbiased estimate of the outcome by generating a sample with a distribution of potential confounders unrelated to exposure, thereby making the results more credible (22).

Therefore, this study investigated the causal relationship between tea consumption habits and cognitive decline by assessing cumulative exposure through refined measurements, and further validated the findings using the IPTW method.

## 2 Methods

### 2.1 Study participants

The data used in this study came from the Chinese Longitudinal Healthy Longevity Survey (CLHLS), an ongoing, nationwide longitudinal study. The CLHLS employed multistage, stratified cluster sampling to randomly select counties and cities in 23 of 31 provinces, roughly covering more than 85% of the Chinese population. Beginning in 1998, CLHLS conducted eight waves of surveys, with follow-up surveys every 2 to 3 years (wave 1 to 8: 1998, 2000, 2002, 2005, 2008, 2011, 2014, and 2018). More details of the study have been published elsewhere (23, 24). The survey was conducted through face-to-face interviews with structured questionnaires completed by trained professional investigators.

Because participants’ heights were measured beginning in 2008, this study used 4 waves of longitudinal data from 2008, 2011, 2014, and 2018 years, with 2008 year as the baseline. Participants were included if they (1) had at least one follow-up visit, (2) had complete tea and cognition information at baseline and last survey, and (3) had no cognitive impairment at baseline. Due to the small percentage of missing key covariates, participants with missing data were directly excluded. Ultimately, this study included 6,641 participants aged 60 years and older. Supplementary Figure 1 demonstrates the detailed flowchart of the subjects in the study.

### 2.2 Tea consumption habits

**Figure 1:**
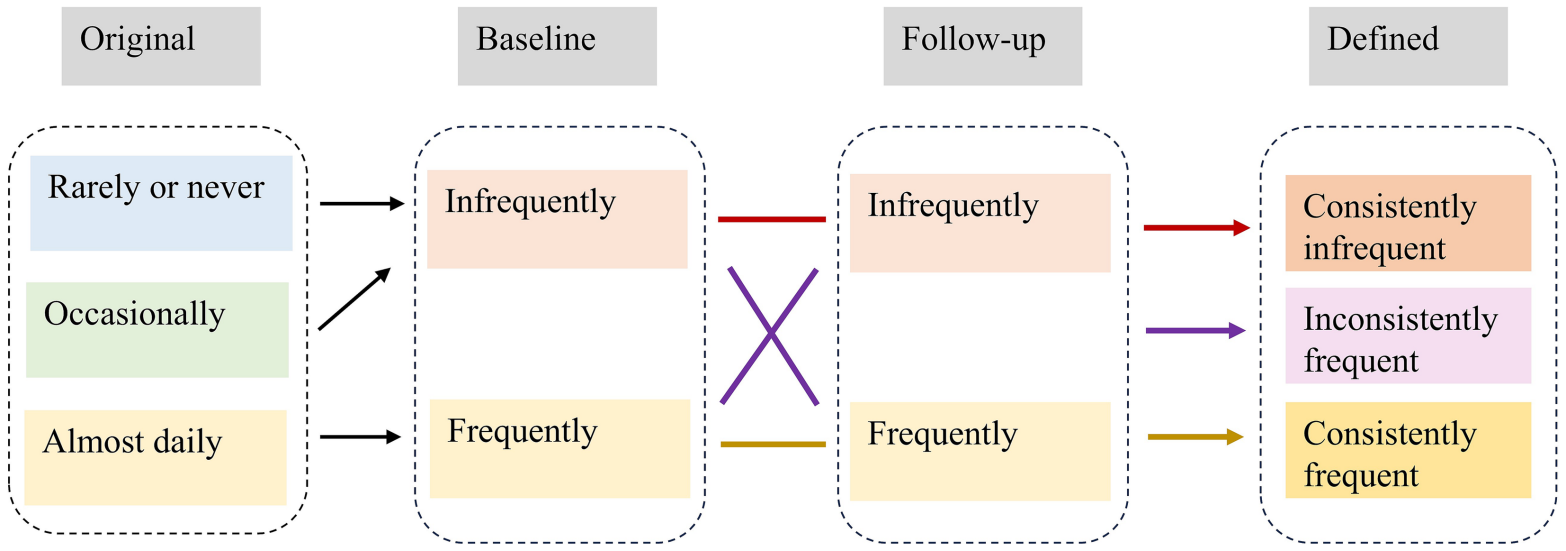
Definitional conversion of tea consumption habit.

Each survey collected information on the current frequency of tea drinking. The response was categorized into three groups: almost daily (≥1 cup/day), occasionally (<1 cup/day but ≥1 cup/month), and rarely or never (<1 cup/month or never drinks tea) (25). To further reflect the uniformity of tea consumption habits in subsequent years, we created a new variable. Tea consumption frequency was first divided into frequently (almost daily) and infrequently (occasionally and rarely or never). The frequency was then categorized into three groups based on the frequency of tea consumption at baseline and last follow-up survey (26). Participants were defined as “consistently infrequent drinkers” if they drank tea infrequently both at baseline and last follow-up survey, which served as the reference group in the analysis. In contrast, they were defined as “consistently frequent drinkers” if they drank frequently both at baseline and last follow-up. In between, they were defined as “inconsistently frequent drinkers” if they drank tea frequently/infrequently at baseline, but infrequently/frequently at last follow-up survey. The relevant definitional transitions are shown in Figure 1. The study used 2008 as the baseline and followed participants until the following occurred: cognitive decline, loss to follow-up, death, or study endpoint, whichever came first.

### 2.3 Cognitive function

Cognitive function was assessed by the Mini-Mental State Examination (MMSE) at baseline and each follow-up survey. This is an internationally standardized assessment tool for use in clinical cognitive screening tests (27). The Chinese version of the MMSE has been adapted for Chinese and has been shown to be valid and reliable in previous studies (24). The 24-item questionnaire assessed cognitive function across five dimensions: orientation, registration, attention and calculation, recall, and language. The total score ranges from 0 to 30, with higher scores representing better cognitive function (28). We defined the “Unable to answer” as “fault” (29). Based on previous literature, individuals with a baseline MMSE score of 18 or less were excluded, as they were considered to have severe cognitive impairment (30, 31). Cognitive decline was defined as a reduction of more than 3 points at follow-up (32, 33).

### 2.4 Covariates

Based on previous studies, we selected sociodemographic characteristics, lifestyle, and health status at baseline as potential confounders (14, 18, 28, 29). Covariates included age (continuous), gender (male/female), residence (urban/rural), education level (years of schooling: 0 year/ ≥1 year), household income (< 10,000/10,001–50,000/> 50,000 yuan), living state [living with members/living alone), body mass index (BMI, kg/m^2^; underweight[< 18.5]/normal[18.5–23.9]/overweight[24–27.9]/obese[≥ 28] (34)], current smoking status (yes/no), current drinking alcohol status (yes/no), current exercising status (yes/no), vegetable intake (daily or frequent intake/occasional or rare intake), fruit intake (daily or frequent intake/occasional or rare intake), and self-reported history of chronic diseases: hypertension (with/without/unknown), diabetes (with/without/unknown). In addition, baseline MMSE scores were considered.

### 2.5 Statistical analyses

Subjects were grouped according to the habits of tea consumption during the follow-up period. The characteristics were compared by using chi-square analysis for categorical variables and analysis of variance (ANOVA) for continuous variables. Cox proportional hazard regression models were performed to calculate hazard ratios (HRs) and 95% confidence intervals (CIs) for the association between tea consumption habits and risk of developing cognitive decline. Survival time was defined as the time from baseline to the date of the first occurrence of cognitive decline, loss to follow-up, or study endpoint, whichever came first. The median follow-up time was calculated by the reverse Kaplan–Meier method. The cumulative incidence curves were estimated by Kaplan–Meier method, and differences were tested by log-rank tests. We fitted four models: Model 1 was the unadjusted model; Model 2 adjusted for age (continuous), gender, BMI and residence; Model 3 further controlled for education level, household income, living state, exercising, vegetables and fruit intake based on model 2; Model 4 additionally adjusted for smoking, drinking, hypertension, diabetes, and baseline MMSE score based on model 3.

To obtain more robust results, inverse probability of treatment weighting (IPTW) based on propensity score was used. This method achieves some of the characteristics of a randomized controlled experiment by recalculating individual weights to create a pseudo-population that is almost perfectly balanced (21, 22). Propensity scores were computed using the generalized boosting model (GBM) of the “twang” package in R software (35). We weighted the variables that remained different (*p* < 0.05) after multivariate regression analysis in the cohort (36). Standardized mean differences (SMDs) were used to assess the balance of the covariates before and after weighting, and they were considered well balanced with absolute values less than 0.1 (22). Doubly robust Cox proportional hazard regression models, incorporating relevant covariates and weights, were used to assess the adjusted effect of tea consumption on cognitive decline.

To further investigate potential effect modification among different subgroups, stratified analyses by age, gender, education, fruit intake, and BMI were conducted within the IPTW-adjusted regression models, as well as the interaction between tea consumption habits and these subgroups. In addition, several sensitivity analyses were implemented based on IPTW adjustment before and after: (1) excluding participants with baseline cognitive scores of 3 or lower (because they were less likely to have experienced cognitive decline); (2) using a decrease in cognitive scores of more than 6 points at follow-up to define cognitive decline (33); (3) excluding participants with cognitive impairment defined as baseline cognitive scores of 24 or less (28); (4) Sleep quality (good/fair/bad) was additionally adjusted. All statistical analyses were performed using R software (version 4.4.0), with two-tailed *p* < 0.05 indicating statistical significance.

## 3 Results

### 3.1 Participant characteristics

Table 1 shows the baseline characteristics of participants by tea consumption habits. Overall, a total of 6,641 participants were included, with a mean age of 80.54 (SD: 10.23) years at baseline, and 49.51% were male. Compared to infrequent tea drinkers, frequent tea drinkers, especially consistent frequent tea drinkers, had higher cognitive scores. Inconsistently frequent and consistently frequent tea drinkers were more likely to be younger, male, educated, exercise regularly, live in urban area with members, have higher incomes, and consume vegetables and fruits frequently, compared to consistently infrequent tea drinkers. However, they were also more likely to smoke, drink alcohol, and have a higher prevalence of hypertension and diabetes. In addition, during a median follow-up period of 6.2 (95% CI: 6.1, 11.2) years, 3,177 (47.8%) participants with normal cognitive function at baseline developed cognitive decline, with a corresponding incidence density of 85.5 (95% CI: 78.8, 98.2) per 1,000 person-years (Table 2). There were 1937 (51.5%), 901 (46.0%) and 339 (36.6%) cognitive decline events among consistent infrequent, inconsistent frequent and consistent frequent tea drinkers, respectively, with corresponding incidence densities of 92.6 (95% CI: 83.3, 101.9), 81.6 (95% CI: 69.5, 93.7), and 65.3 (95% CI: 49.3, 81.2) per 1,000 person-years.

**Table 1:** Baseline characteristics of participants by tea consumption habits.

Variable^a^	Consistently infrequent drinker (*n* = 3,758)	Inconsistently frequent drinker (*n* = 1,957)	Consistently frequent drinker (*n* = 926)	*p*
Baseline MMSE score [mean (SD)]	2,671 (3.1)	2,720 (2.9)	2,769 (2.7)	<0.001
Age [mean (SD)]	8,122 (10.4)	7,997 (9.8)	7,901 (10.0)	<0.001
Age group				<0.001
<80	1,678 (44.7)	970 (49.6)	491 (53.0)	
80–90	1,186 (31.6)	603 (30.8)	274 (29.6)	
>90	894 (23.8)	384 (19.6)	161 (17.4)	
Gender				<0.001
Male	1,492 (39.7)	1,130 (57.7)	666 (71.9)	
Female	2,266 (60.3)	827 (42.3)	260 (28.1)	
Residence				<0.001
Urban	1,286 (34.2)	852 (43.5)	434 (46.9)	
Rural	2,472 (65.8)	1,105 (56.5)	492 (53.1)	
Living state				0.019
With members	3,102 (82.5)	1,642 (83.9)	799 (86.3)	
Alone	656 (17.5)	315 (16.1)	127 (13.7)	
Smoking				<0.001
No	3,130 (83.3)	1,448 (74.0)	593 (64.0)	
Yes	628 (16.7)	509 (26.0)	333 (36.0)	
Drinking				<0.001
No	3,141 (83.6)	1,488 (76.0)	610 (65.9)	
Yes	617 (16.4)	469 (24.0)	316 (34.1)	
Exercising				<0.001
No	2,472 (65.8)	1,232 (63.0)	523 (56.5)	
Yes	1,286 (34.2)	725 (37.0)	403 (43.5)	
Education level (years)				<0.001
0	2,191 (58.3)	900 (46.0)	327 (35.3)	
≥1	1,567 (41.7)	1,057 (54.0)	599 (64.7)	
Household income (RMB)				<0.001
<10,000	2,052 (54.6)	980 (50.1)	389 (42.0)	
10,001–50,000	1,275 (33.9)	717 (36.6)	395 (42.7)	
>50,000	431 (11.5)	260 (13.3)	142 (15.3)	
BMI (kg/m^2^)				<0.001
Underweight (<18.5)	1,031 (27.4)	428 (21.9)	190 (20.5)	
Normal (18.5–23.9)	2,099 (55.9)	1,162 (59.4)	558 (60.3)	
Overweight (24–27.9)	498 (13.3)	295 (15.1)	147 (15.9)	
Obese (≥28)	130 (3.5)	72 (3.7)	31 (3.3)	
Fruit intake				<0.001
Daily/frequent	1,428 (38.0)	865 (44.2)	450 (48.6)	
Occasional/rare	2,330 (62.0)	1,092 (55.8)	476 (51.4)	
Vegetables intake				0.007
Daily/frequent	3,436 (91.4)	1,791 (91.5)	875 (94.5)	
Occasional/rare	322 (8.6)	166 (8.5)	51 (5.5)	
Hypertension				0.002
With	819 (21.8)	450 (23.0)	203 (21.9)	
Without	2,851 (75.9)	1,485 (75.9)	715 (77.2)	
Unknown	88 (2.3)	22 (1.1)	8 (0.9)	
Diabetes				<0.001
With	96 (2.6)	66 (3.4)	34 (3.7)	
Without	3,584 (95.4)	1,873 (95.7)	885 (95.6)	
Unknown	78 (2.1)	18 (0.9)	7 (0.8)	
Cognitive decline	1,937 (51.5)	901 (46.0)	339 (36.6)	<0.001

^a^Data are presented as number (percentage) of patients unless otherwise indicated.

**Table 2:** Incidence densities of cognitive decline development according to tea consumption habits.

Variables	Total	Consistently infrequent drinker	Inconsistently frequent drinker	Consistently frequent drinker
Participants, *n*	6,641	3,758	1,957	926
Participants, person-years	37157.4	20918.5	11043.3	5195.6
Events, *n*	3,177	1,937	901	339
Incidence density, per 1,000 person-years (95%CI)	85.5 (78.8,98.2)	92.6 (83.3,101.9)	81.6 (69.5, 93.7)	65.3 (49.3,81.2)

### 3.2 Association between tea consumption habits and cognitive decline

**Figure 2:**
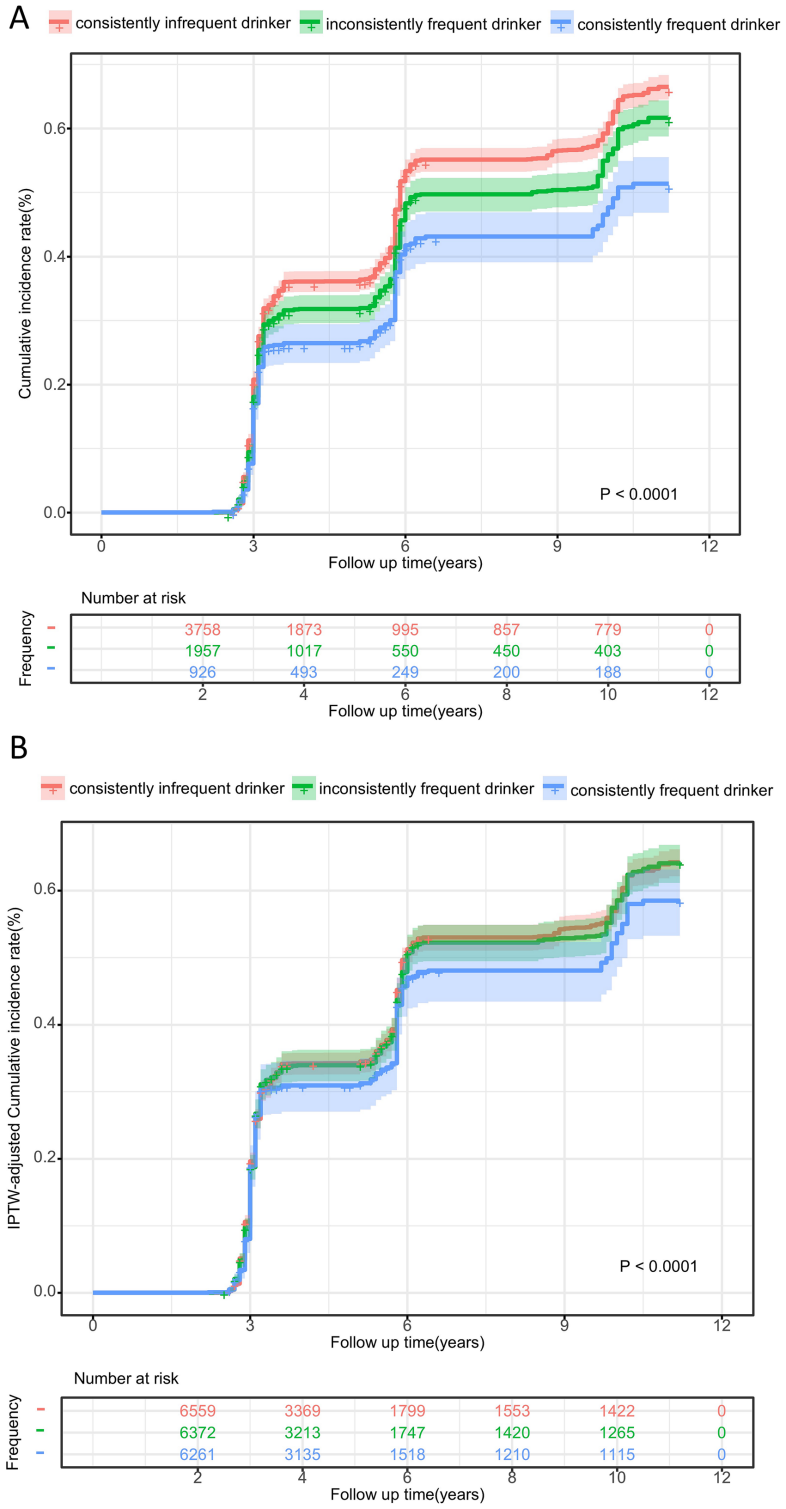
Cumulative incidence curve for cognitive decline according to tea consumption frequency before **(A)** and after IPTW adjustment **(B)**. ^a^The *p*-values was calculated by log-rank test.

**Figure 3:**
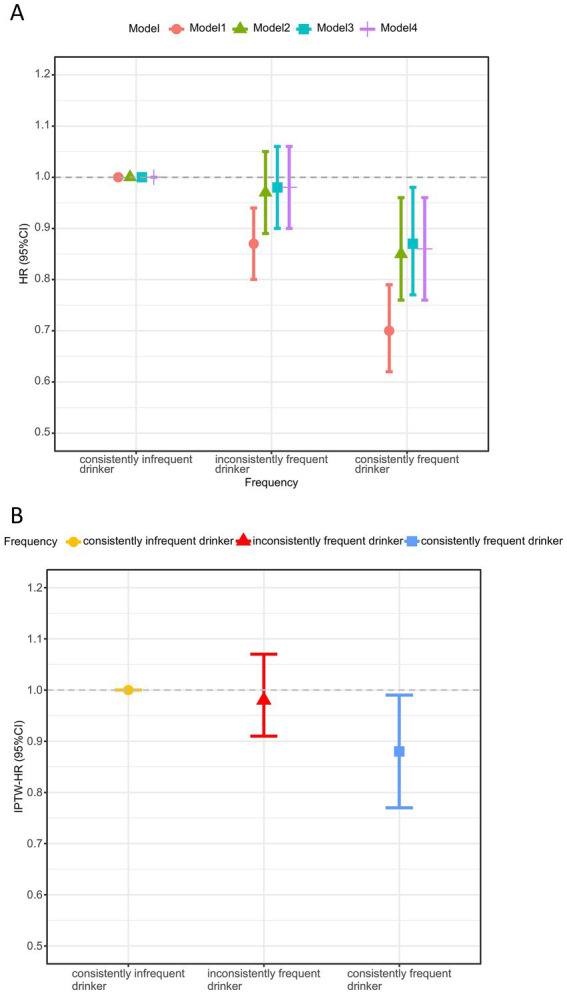
Association between tea consumption habits and cognitive decline before **(A)** and after IPTW adjustment **(B)**^a^. HR, hazard ratio; CI, confidence interval; IPTW, inverse probability of treatment weighting. ^a^Model 1 was unadjusted; Model 2 adjusted for age, gender, BMI and residence; Model 3 additional adjustments for education level, household income, living state, exercising, vegetables and fruit intake upon model 2; Model 4 additional adjustments for smoking, drinking, hypertension, diabetes, and baseline MMSE score upon model 3. The IPTW analysis was based on model 4, adjusting for variables that remain different after multivariate adjustment: age, gender, BMI, education level, exercising, fruit intake, diabetes, baseline MMSE score, and weights.

The cumulative incidence curve indicated that consistently frequent tea drinkers had the lowest incidence of cognitive decline, which gradually increased as tea consumption frequency decreased (log-rank test: *p* < 0.001, Figure 2A). Figure 3A demonstrates that consistent and frequent tea consumption over time was significantly associated with a reduced risk of cognitive decline. In the unadjusted model, inconsistently frequent tea drinkers and consistently frequent tea drinkers had a 13% (HR:0.87, 95% CI: 0.80, 0.94) and 30% (HR: 0.70, 95% CI: 0.62, 0.79) reduced risk of cognitive decline than consistently infrequent drinkers, respectively (Supplementary Table 1). After controlling for sociodemographic, lifestyle, and health status variables, the magnitude of the associations decreased. In fully adjusted models (Model 4), the HR with 95% CI for consistently frequent tea drinkers was 0.86 (95% CI: 0.76, 0.96). However, did not observe an association between inconsistently frequent tea drinkers and cognitive decline. The frequency of tea consumption showed a significant linear trend with cognitive decline (*P* for trend = 0.023). The results suggested that under certain conditions, the higher the frequency of consumption, the stronger the potential protective effect may be. In addition, we found that the relationship between consistently frequent tea consumption and cognitive decline was statistically significant across all four models. Therefore, tea consumption could be considered an independent protective factor against cognitive decline, but it requires maintaining a habit of long-term and frequent tea drinking.

### 3.3 Inverse probability of treatment weighting adjustment

In order to obtain more robust results, we further adjusted for variables that have a potential impact on the effect in the cohorts. Supplementary Table 2 shows the baseline characteristics before and after IPTW adjustment. After adjusting for IPTW, these features were well balanced, and the standardized mean differences (SMD) were all less than 0.1 (Supplementary Figure 2). Figure 2B illustrates the IPTW adjusted cumulative incidence curves between different tea frequencies, which still remained significantly different (adjusted log-rank test: *p* < 0.001).

Maintaining frequent tea consumption over time could reduce the risk of cognitive decline (Figure 3B). Based on the IPTW-adjusted Cox proportional hazards regression model, consistently frequent tea drinkers had a 12% (HR: 0.88, 95% CI: 0.77, 0.99) lower risk of cognitive decline than consistently infrequent tea drinkers (Supplementary Table 3). Similarly, no association was also found between inconsistently frequent tea consumption and cognitive decline. In addition, the risk of cognitive decline decreased with increasing frequency of tea consumption (*P* for trend < 0.001). The above results all indicated that consistent daily tea consumption over a long period, maintaining good consumption habits, could potentially exert protective effects and reduce the risk of cognitive decline.

### 3.4 Subgroup and interaction analysis

**Figure 4:**
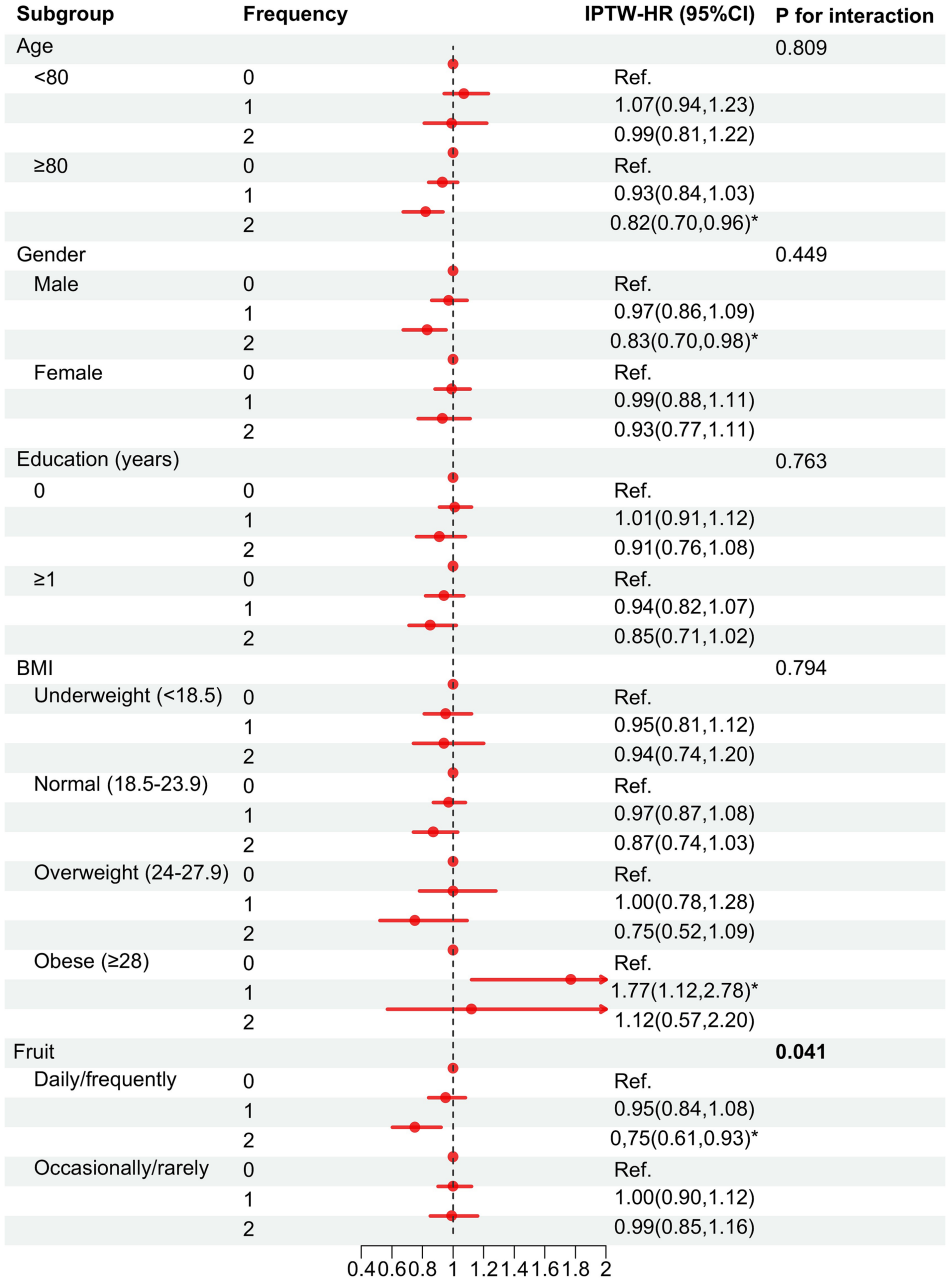
Association between tea consumption habits and cognitive decline in the subgroups after IPTW adjustment^a^. Frequency = 0, consistently infrequent drinker; 1, inconsistently frequent drinker; 2, consistently frequent drinker. HR, hazard ratio; CI, confidence interval. ^a^Based on model 4 adjusted for variables that remain different after multivariate adjustment: age, gender, BMI, education level, exercising, fruit intake, diabetes, baseline MMSE score, and weights. ^*^p < 0.05.

Based on IPTW-multivariate adjusted models, subgroup analyses of age, gender, BMI, education, and fruit intake were performed (Figure 4). The protective effect of tea appeared to be more pronounced in the subgroups of older, male, and regular fruit intake, except for the BMI ≥ 28 kg/m^2^ subgroup. In addition, we found a significant interaction between fruit and tea consumption, suggesting that the protective effect was greater in those who frequently consume both fruit and tea (*P* for interaction: 0.041).

### 3.5 Sensitivity analysis

To obtain more robust findings, multiple sensitivity analyses before and after IPTW adjustment were conducted. Firstly, we excluded participants with baseline cognitive scores of 3 or less (Supplementary Table 4). Second, cognitive decline was defined by a reduction in cognitive scores of more than 6 points at follow-up (Supplementary Table 5). Third, cognitive impairment defined as a baseline cognitive score of 24 or lower was excluded (Supplementary Table 6). Finally, sleep quality was included (Supplementary Table 7). Similar results were obtained before and after the corresponding IPTW adjustment.

## 4 Discussion

In this large population study of Chinese adults aged 60 and older, we found that consistently maintaining frequent tea consumption reduced the risk of cognitive decline during 10 years of follow-up, whereas inconsistently frequent tea consumption did not. This protective effect remained robust after adjusting for IPTW. In addition, the interaction analysis revealed that frequent intake of tea and fruit had a synergistic effect on reducing cognitive decline.

Ng et al. revealed that higher frequency of tea consumption was associated with lower risk of cognitive decline among Chinese Singaporeans (18). In a prospective study implemented by Li et al. with a one-year follow-up, it was shown that drinking tea was associated with a lower incidence of cognitive impairment, and prevented declines in memory and associative learning by affecting the volume of the posterior corpus callosum among Chinese older adults (14). Both Noguchi-Shinohara et al. and Shirai et al. (16, 17) suggested that green tea consumption was associated with a reduced risk of cognitive decline in Japanese older adults. These findings are partially consistent with the present study.

It is worth noting that previous studies have only considered baseline tea consumption information and ignored changes in tea drinking habits. Therefore, the present study developed a new measurement method that assessed cumulative exposure using baseline and last follow-up tea consumption information and consistency, to identify changes in tea drinking habits. The study suggested that consistently frequent tea consumption could reduce the risk of cognitive decline, whereas inconsistent and frequent tea consumption did not. This suggested that the protective effect of tea on cognitive function may be realized in the form of long-term accumulation. In other words, it is more likely to benefit from drinking tea every day without interruption for a long period of time. This finding provided a more precise and reliable conclusion on the effect of tea consumption frequency on cognitive decline, and helps to advance the field of research focusing on the dose of tea intake.

In contrast, a prospective study conducted in Shanghai, China, did not find an association between tea consumption and cognitive decline (15). The reasons for this heterogeneity may be due to the relatively short follow-up period of just 2 years, during which the benefits of tea consumption may not have manifested. Furthermore, previous research was solely based on the urban community in Shanghai, whereas the current study was a nationwide survey that included both urban and rural areas. In addition, two prospective studies in the United States and Germany reported no effect of black and green tea consumption on cognitive decline (19, 20). Direct comparisons could not be made because the baseline survey for the present study did not have information on the type of tea consumption. In addition, there may have been differences in genetic exposure and tea brewing methods. In Chinese culture, tea is traditionally consumed without milk or sugar, whereas in the West, the addition of additives is common (19). Studies have shown that tea polyphenols interact with milk proteins, especially caseins, to reduce the antioxidant activity of tea (37).

In addition, we found tea consumption appeared to be detrimental to cognitive function in the obese subgroup (BMI ≥ 28 kg/m^2^). Studies have shown that obesity indices such as BMI, waist circumference, and waist-to-hip ratio were all associated with poorer performance on multiple tests of cognitive function, and there was a cumulative effect among them (38, 39). Generally, obese individuals have multiple obesity indices above the normal range with cumulative effects, and the cognitive benefits of tea may not be enough to offset these deficits. In addition, we also found an interaction between fruit and tea intake. Regular consumption of both fruit and tea has a greater protective effect on cognitive health. A study suggested daily fruit intake reduced risk of cognitive impairment in older Chinese adults (40). A multicenter study from Europe also suggested that regular fruit intake improves cognitive and mental health (41). A study has shown that flavanol-rich extract prepared from lychee fruit and green tea could reduce progressive cognitive and molecular deficits in a triple-transgenic animal model of Alzheimer disease (42). In addition, early-stage AD has been found to be associated with high insulin concentrations in response to glucose challenge (hyperinsulinaemia) in combination with reduced insulin-mediated glucose uptake (insulin resistance) (43). Tea and fruit may affect cognitive function by influencing blood sugar levels and insulin. Therefore, regular intake of tea and fruit may enhance the benefits on cognitive function.

Results from multifactorial Cox proportional risk modeling based on the 2008–2018 cohort have shown that consistently frequent tea consumption reduced the risk of cognitive decline, whereas inconsistently frequent tea consumption was not significant. Due to the non-random assignment of study participants in observational studies, there is selection bias and significant differences in baseline characteristics between groups. In order to reduce selection bias to obtain more reliable results, we used propensity score method. The propensity score methods aim to simulate randomization, attenuate the likelihood of confounding in non-randomized observational studies (21). IPTW was chosen because it uses the entire dataset, increasing the effective sample size (21). Therefore, we further adjusted all covariates that could confound the association after multivariate regression in the cohort and important covariates, such as age and gender. In fact, they themselves were significant. After IPTW adjustment, all covariates were balanced with the standardized mean difference being less than 0.1. The protective effect of tea consumption against cognitive decline remained robust. All results indicated that the protective effect of tea against cognitive decline requires a long-term commitment to drinking tea daily and maintaining an unbroken habit.

As mentioned earlier, the neuroprotective mechanisms of tea are complex. A growing number of studies have explored the potential mechanisms of tea’s effects on neurodegenerative diseases (7). Studies have shown that excessive reactive oxygen species (ROS) production and accumulation of defective mitochondria in brain cells, were associated with neurodegenerative diseases. Whereas catechins, especially epigallocatechin gallate (EGCG), were strong antioxidants that scavenge free radicals (44). Administration of catechins to rats prevented amyloid-induced cognitive impairment and reduced lipid peroxide and ROS levels in both hippocampus and plasma by 20% (45). In addition, it involves the modulation of cell signaling pathways and metal chelator activity (7). Theaflavins, which are mainly found in black tea, can protect neuronal cells (PC12) from H_2_O_2_-induced apoptosis and exert neuroprotective effects (46). In addition, caffeine is known for its effects on attention, improving cognitive and physical function by blocking adenosine receptors (47).

Cognitive impairment may also affect eating habits. Studies have shown that low levels of blood carotenoids were found among Alzheimer’s disease (AD) patients compared to healthy controls (48). Therapeutic supplements of carotenoids have been proposed as an alternative treatment and prevention strategy for AD (48). Antioxidant therapy has been explored as a new approach to the prevention and treatment of neurodegenerative diseases in which oxidative stress was a contributing factor (49). Natural antioxidants include enzymatic and non-enzymatic antioxidants, such as polyphenols, vitamins, minerals and carotenoids (49). Tea is rich in non-enzymatic antioxidants and may be recommended as an effective food for people with early-stage dementia. In addition, dietary treatments have been proposed (50).

This study has several strengths. First, instead of relying solely on baseline tea consumption information, we developed a more refined measurement method, considering that tea drinking habits may change over time, which was more conducive in clarifying the causal relationship between tea consumption and cognitive decline. It indicated that the protective effect of tea on cognitive function is realized through long-term accumulation, and promoted a focus on cumulative exposure. Second, we controlled for as many confounders as possible, including using traditional multivariate adjustments and IPTW adjustments, and the results remained robust. Last, this study was based on a nationally representative cohort of older adults with a large sample size and a long follow-up period. It has favorable applicability in China.

There are also several limitations in this study. First, unmeasured and residual confounders may still be present. Second, tea consumption frequency was self-reported by participants, recall bias may exist. Third, although the MMSE has been well validated in assessing cognition, there was a lack of pathophysiologic assessment of cognitive function in the community-based survey. Some studies have used 28 or 24 points as the initial cut-off point with 2 or 6 points per decline as the threshold for moderate and severe cognitive impairment, respectively (51, 52). Therefore, in this study, we used 3 and 6 points as thresholds to reduce the possibility of misclassification, respectively. More clinical trials are needed to validate the results of this study. Finally, due to lack of data, we could not obtain the exact quantity and type of tea intake. Green tea contains rich catechins, while in black, oolong and dark teas catechins are converted to theaflavins, thearubigins and theabrownins (53). Different types of tea have varying bioactive components, which may have varying effects on cognitive decline. More information needs to be collected in the future for further research.

## 5 Conclusion

Long-term consistently frequent tea consumption was associated with a reduced risk of cognitive decline in older Chinese adults, while inconsistently frequent tea consumption was not associated, even after IPTW adjustment. In addition, there may be a synergistic effect between regular tea drinking and fruit consumption on cognitive health. Advocating long-term frequent tea consumption and maintaining an unbroken habit could promote neurocognitive health.

## Data Availability

The original contributions presented in the study are included in the article/Supplementary material, further inquiries can be directed to the corresponding authors.
