# Supplementary material for: Maintaining long-term frequent tea consumption could reduce the risk of cognitive decline: results from a 10-year longitudinal study
Source: Front Nutr. 2025 Jun 18;12:1569850. doi: 10.3389/fnut.2025.1569850 (PMC12218255; doi:10.3389/fnut.2025.1569850)
Supplement: Supplementary file 1 [file Table_1.docx]

**Supplementary Material:**

**Supplementary Figure 1.** The detailed flowchart of the participants in this study.

**Supplementary Figure 2** Standardized mean differences (SMD) of covariates before and after applying inverse probability of treatment weighting (IPTW).

**Supplementary Table 1.** Association between tea consumption habits and cognitive decline.

**Supplementary Table 2.** Baseline characteristics of participants according to tea consumption habits with IPTW adjusted.

**Supplementary Table 3.** Association between tea consumption habits and cognitive decline after IPTW adjustment.

**Supplementary Table 4.** Sensitivity analyses by excluding individuals with baseline MMSE scores of 3 or less, before and after IPTW adjustment.

**Supplementary Table 5.** Sensitivity analyses by defining cognitive decline as a decrease of more than 6 points at follow-up, before and after IPTW adjustment.

**Supplementary Table 6.** Sensitivity analyses by excluding individuals with baseline MMSE scores of 24 or less, before and after IPTW adjustment.

**Supplementary Table 7.** Sensitivity analyses by additionally adjusting sleep quality variable before and after IPTW adjustments.


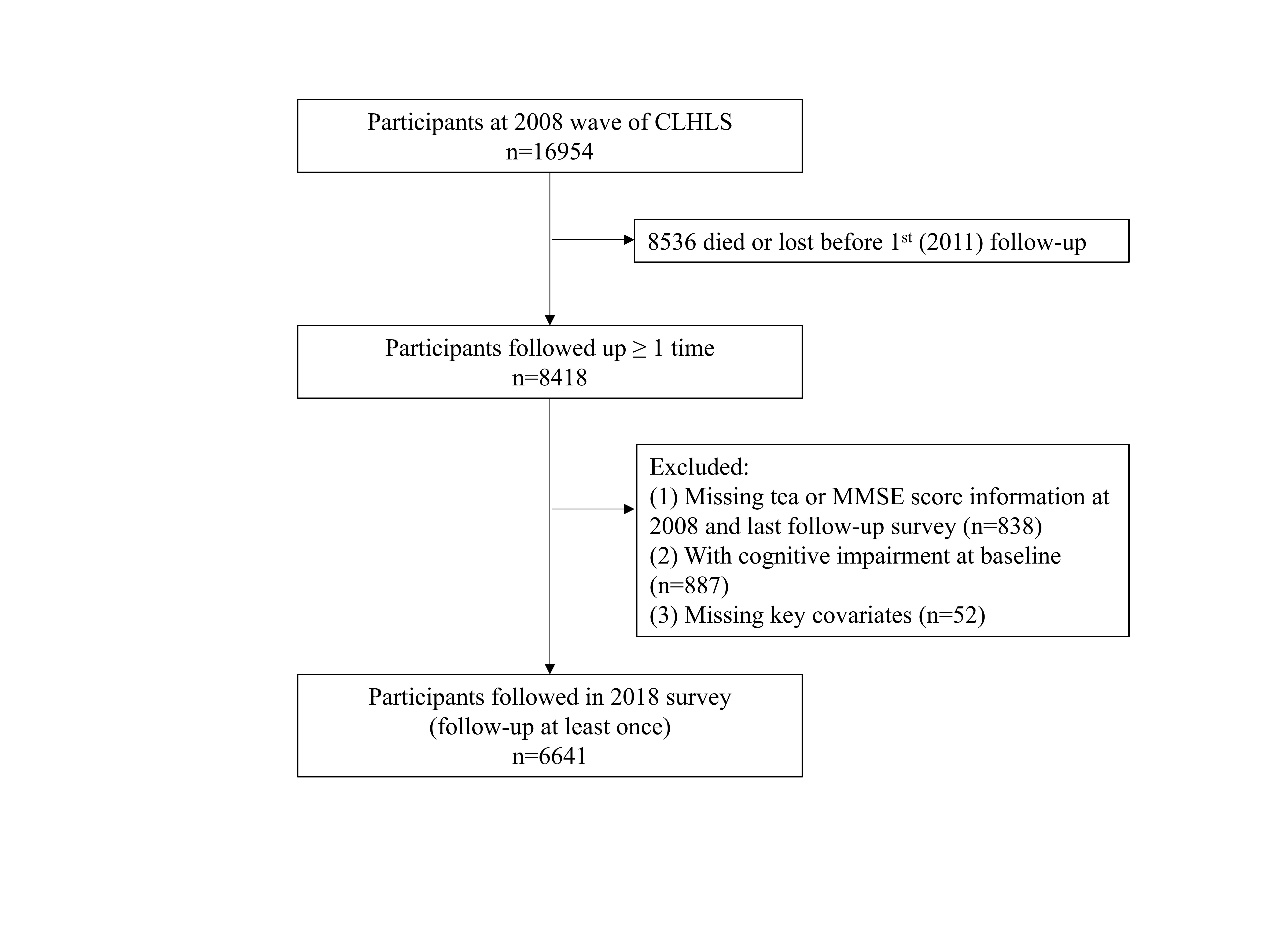


**Supplementary Figure 1.** The detailed flowchart of the participants in this study.

**
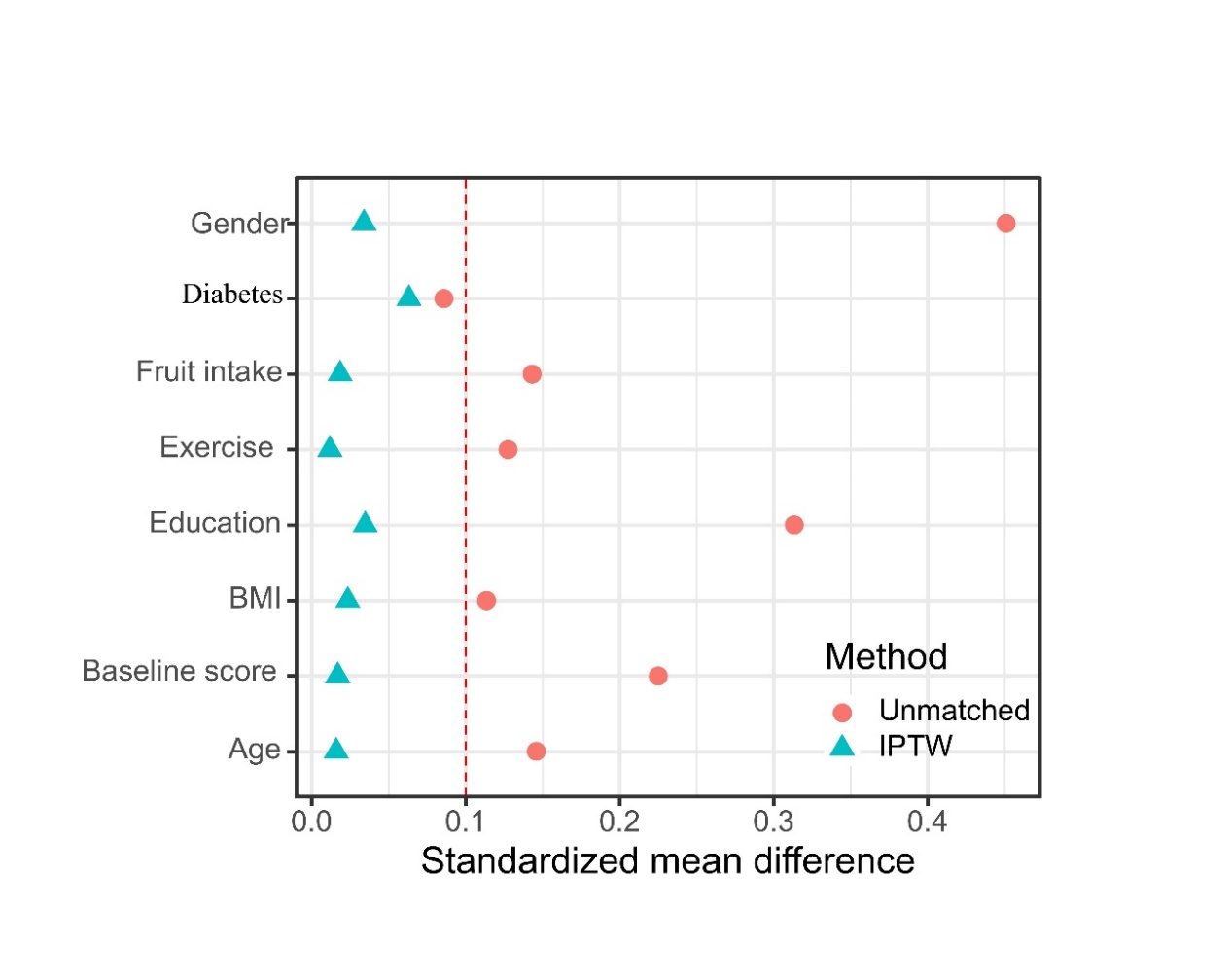
**

**Supplementary Figure 2.** Standardized mean differences (SMD) of covariates before and after applying inverse probability of treatment weighting (IPTW).

**Supplementary Table 1.**

Association between tea consumption habits and cognitive decline.

| Frequency | Hazard Ratios (95%CI) | | | | | | | |
| --- | --- | --- | --- | --- | --- | --- | --- | --- |
|  | Model 1^a^ | | Model 2^b^ | | Mode l3^c^ | | Model 4^d^ | |
| Consistently  infrequent drinker | reference | | reference | | reference | | reference | |
| Inconsistently  frequent drinker | 0.87(0.80,0.94) | <0.001 | 0.97(0.89,1.05) | 0.442 | 0.98(0.90,1.06) | 0.627 | 0.98(0.90,1.06) | 0.572 |
| Consistently  frequent drinker | 0.70(0.62,0.79) | <0.001 | 0.85(0.76,0.96) | 0.009 | 0.87(0.77,0.98) | 0.019 | 0.86(0.76,0.96) | 0.011 |
| P for trend ^e^ |  | <0.001 |  | 0.032 |  | 0.056 |  | 0.023 |

^a^: Model 1was unadjusted;

^b^: Model 2 adjusted for age, gender, BMI and residence;

^c^: Model 3 additional adjustments for education level, household income, living state, exercising, vegetables and fruit intake upon model 2;

^d^: Model 4 additional adjustments for smoking, drinking, hypertension, diabetes, and baseline MMSE score upon model 3.

^e^: Trend tests were performed by including the ordinal variable in the regression analysis.

**Supplementary Table 2.**

Baseline characteristics of participants according to tea consumption habits with IPTW adjusted.

| Variable ^a^ | Unmatched | | | | | IPTW ^b^ | | | | |
| --- | --- | --- | --- | --- | --- | --- | --- | --- | --- | --- |
|  | Consistently  Infrequent drinker  (n=3758) | Inconsistently  frequent drinker  (n=1957) | Consistently  frequent drinker  (n=926) | P | SMD | Consistently  Infrequent drinker  (n=6558.85) | Inconsistently  frequent drinker  (n=6372.19) | Consistently  frequent drinker  (n=6260.60) | P | SMD |
| Age (mean (SD)) | 81.22 (10.40) | 79.97 (9.89) | 79.01 (10.00) | <0.001 | 0.146 | 80.54 (10.20) | 80.45 (10.01) | 80.69 (10.22) | 0.864 | 0.016 |
| Gender |  |  |  | <0.001 | 0.451 |  |  |  | 0.395 | 0.034 |
| Male | 1492 (39.7) | 1130 (57.7) | 666 (71.9) |  |  | 3223.5 (49.1) | 3189.5 (50.1) | 3236.6 (51.7) |  |  |
| Female | 2266 (60.3) | 827 (42.3) | 260 (28.1) |  |  | 3335.3 (50.9) | 3182.6 (49.9) | 3024.0 (48.3) | |  |
| Exercising |  |  |  | <0.001 | 0.128 |  |  |  | 0.85 | 0.012 |
| No | 2472 (65.8) | 1232 (63.0) | 523 (56.5) |  |  | 4183.9 (63.8) | 4076.8 (64.0) | 4047.4 (64.6) |  |  |
| Yes | 1286 (34.2) | 725 (37.0) | 403 (43.5) |  |  | 2374.9 (36.2) | 2295.4 (36.0) | 2213.2 (35.4) | |  |
| Education level (years) |  |  |  | <0.001 | 0.313 |  |  |  | 0.36 | 0.035 |
| 0 | 2191 (58.3) | 900 (46.0) | 327 (35.3) |  |  | 3396.8 (51.8) | 3257.2 (51.1) | 3078.6 (49.2) |  |  |
| ≥1 | 1567 (41.7) | 1057 (54.0) | 599 (64.7) |  |  | 3162.1 (48.2) | 3115.0 (48.9) | 3182.0 (50.8) | |  |
| BMI (kg/m^2^) |  |  |  | <0.001 | 0.114 |  |  |  | 0.979 | 0.024 |
| Underweight (<18.5) | 1031 (27.4) | 428 (21.9) | 190 (20.5) |  |  | 1631.4 (24.9) | 1588.3 (24.9) | 1507.7 (24.1) |  |  |
| Normal (18.5-23.9) | 2099 (55.9) | 1162 (59.4) | 558 (60.3) |  |  | 3779.6 (57.6) | 3682.5 (57.8) | 3699.4 (59.1) | |  |
| Overweight (24-27.9) | 498 (13.3) | 295 (15.1) | 147 (15.9) |  |  | 920.4 (14.0) | 878.4 (13.8) | 856.7 (13.7) | |  |
| Obese (≥28) | 130 (3.5) | 72 (3.7) | 31 (3.3) |  |  | 227.5 (3.5) | 222.9 (3.5) | 196.7 (3.1) |  |  |
| Fruit intake |  |  |  | <0.001 | 0.143 |  |  |  | 0.705 | 0.019 |
| Daily/frequent | 1428 (38.0) | 865 (44.2) | 450 (48.6) |  |  | 2696.7 (41.1) | 2638.1 (41.4) | 2660.0 (42.5) |  |  |
| Occasional/rare | 2330 (62.0) | 1092 (55.8) | 476 (51.4) |  |  | 3862.2 (58.9) | 3734.1 (58.6) | 3600.6 (57.5) | |  |
| Diabetes |  |  |  | <0.001 | 0.086 |  |  |  | 0.152 | 0.063 |
| With | 96 (2.6) | 66 (3.4) | 34 (3.7) |  |  | 190.6 (2.9) | 187.4 (2.9) | 178.0 (2.8) |  |  |
| Without | 3584 (95.4) | 1873 (95.7) | 885 (95.6) |  |  | 6267.0 (95.6) | 6107.4 (95.8) | 6046.1 (96.6) | |  |
| Unknown | 78 (2.1) | 18 (0.9) | 7 (0.8) |  |  | 101.3 (1.5) | 77.4 (1.2) | 36.5 (0.6) |  |  |
| Baseline MMSE score  (mean (SD)) | 26.71 (3.12) | 27.20 (2.96) | 27.69 (2.72) | <0.001 | 0.225 | 27.00 (3.02) | 27.04 (3.00) | 27.08 (3.02) | 0.804 | 0.017 |

Abbreviations: IPTW: inverse probability of treatment weighting; SMD: standardized mean difference.

^a^: Data are presented as number (percentage) of patients unless otherwise indicated.

^b^: A dummy population with balanced covariates generated by weighting the probability of individuals receiving treatment.

**Supplementary Table 3.**

Association between tea consumption habits and cognitive decline after IPTW adjustment.

| Frequency | IPTW-HR (95%CI) ^a^ | P |
| --- | --- | --- |
| consistently infrequent drinker | reference | |
| inconsistently frequent drinker | 0.98(0.91,1.07) | 0.695 |
| consistently frequent drinker | 0.88(0.77,0.99) | 0.048 |
| P for trend ^b^ |  | <0.001 |

Abbreviations: IPTW: inverse probability of treatment weighting; HR: hazard ratio; CI: confidence interval.

^a^: based on model 4 adjusted for variables that remain different after multivariate adjustment: age, gender, BMI, education level, exercising, fruit intake, diabetes, baseline MMSE score, and weights.

^b^: Trend tests were performed by including the ordinal variable in a linear regression analysis.

**Supplementary Table 4**

Sensitivity analyses by excluding individuals with baseline MMSE scores of 3 or less, before and after IPTW adjustments.

|  | consistently infrequent  drinker | inconsistently frequent  drinker | | consistently frequent  drinker | |
| --- | --- | --- | --- | --- | --- |
| HR (95%CI) ^a^ | reference | 0.96(0.89,1.04) | 0.347 | 0.85(0.75,0.95) | 0.005 |
| IPTW-HR (95%CI) ^b^ | reference | 0.96(0.89,1.04) | 0.375 | 0.86(0.75,0.98) | 0.022 |

Abbreviations: IPTW: inverse probability of treatment weighting; HR: hazard ratio; CI: confidence interval.

^a^: based on model 4 adjusted for age, gender, BMI, residence, education level, household income, living state; exercising, vegetables and fruit intake, smoking, drinking, hypertension, diabetes, and baseline MMSE score.

^b^: based on model 4 adjusted for variables that remain different after multivariate adjustment: age, gender, BMI, education level, exercising, fruit intake, baseline MMSE score, and weights.

**Supplementary Table 5**

Sensitivity analyses by defining cognitive decline as a decrease of more than 6 points at follow-up, before and after IPTW adjustments.

|  | consistently infrequent  drinker | inconsistently frequent  drinker | | consistently frequent  drinker | |
| --- | --- | --- | --- | --- | --- |
| HR (95%CI) ^a^ | reference | 0.90(0.81,0.99) | 0.028 | 0.74(0.63,0.66) | <0.001 |
| IPTW-HR (95%CI) ^b^ | reference | 0.90(0.81,0.99) | 0.034 | 0.77(0.65,0.91) | 0.002 |

Abbreviations: IPTW: inverse probability of treatment weighting; HR: hazard ratio; CI: confidence interval.

^a^: based on model 4 adjusted for age, gender, education level, household income, residence, living state; BMI, smoking, drinking, exercising, vegetables and fruit intake, hypertension, diabetes and baseline MMSE score.

^b^: based on model 4 adjusted for variables that remain different after multivariate adjustment: age, gender, BMI, education level, exercising baseline MMSE score, and weights.

**Supplementary Table 6**

Sensitivity analyses by excluding individuals with baseline MMSE scores of 24 or less before and after IPTW adjustments.

|  | consistently infrequent  drinker | inconsistently frequent  drinker | | consistently frequent  drinker | |
| --- | --- | --- | --- | --- | --- |
| HR (95%CI) ^a^ | reference | 1.02(0.93,1.12) | 0.664 | 0.87(0.76,0.99) | 0.030 |
| IPTW-HR (95%CI) ^b^ | reference | 1.02(0.93,1.12) | 0.676 | 0.87(0.75,0.99) | 0.049 |

Abbreviations: IPTW: inverse probability of treatment weighting; HR: hazard ratio; CI: confidence interval.

^a^: based on model 4 adjusted for age, gender, BMI, residence, education level, household income, living state; exercising, vegetables and fruit intake, smoking, drinking, hypertension, diabetes, and baseline MMSE score.

^a^: based on model 4 adjusted for variables that remain different after multivariate adjustment: age, gender, BMI, education level, exercising, fruit intake, diabetes, baseline MMSE score, and weights

**Supplementary Table 7**

Sensitivity analyses by additionally adjusting sleep quality variable before and after IPTW adjustments.

|  | consistently infrequent  drinker | inconsistently frequent  drinker | | consistently frequent  drinker | |
| --- | --- | --- | --- | --- | --- |
| HR (95%CI) ^a^ | reference | 0.98(0.90,1.06) | 0.552 | 0.86(0.76,0.96) | 0.010 |
| IPTW-HR (95%CI) ^b^ | reference | 0.98(0.90,1.07) | 0.680 | 0.88(0.77,0.99) | 0.046 |

Abbreviations: IPTW: inverse probability of treatment weighting; HR: hazard ratio; CI: confidence interval.

^a^: based on model 4 adjusted for age, gender, BMI, residence, education level, household income, living state; exercising, vegetables and fruit intake, smoking, drinking, hypertension, diabetes, baseline MMSE score and sleep quality.

^a^: based on model 4 adjusted for variables that remain different after multivariate adjustment: age, gender, BMI, education level, exercising, fruit intake, diabetes, baseline MMSE score, and weights
